# The Metric System in Medicine

**Published:** 1904-02-06

**Authors:** C. O. Hawthorne


### The Metric System in Medicine.

By C. O. Hawthorne, M.D., M.R.C.P.

The merits and advantages of a decimal system of weights and measures are recognised with an almost unanimous voice and are regularly advanced as arguments in favour of the adoption of the metric standards in the practical concerns of life. There has even existed for some years an organised association to attain this end; in various schemes of education and examination a knowledge of the metric system is made compulsory; and active opposition to the proposal to replace the existing arbitrary and artificial standards by the simple and scientific decimal method can hardly be said to exist. Yet so effective is the inertia of habit and custom that a reform concerning which nearly all men speak well remains stagnant and unfruitful. According to a recent report the approaching session of Parliament is to witness the introduction in the House of Lords of a Bill to render the adoption of the metric system compulsory throughout the United Kingdom. The educative value of such a proceeding need not be disputed, but it is obvious that it can have little or no effect in compelling the habits of the people. These are fixed by long continued and daily repeated precedents, and men can no more be made to calculate in decimal terms than they can be educated in virtuous motives by the powers of an Act of Parliament. The Bill even if passed would be of no avail against the passive resistance of the great mass of the people. It may be that in some more or less remote future such a law may be successfully put into operation. But in order that this may be possible the mental habits of the people must be prepared by a scheme and system of education which renders metric terms the normal and appropriate symbols of their thoughts. At present these terms and the facts they express are mere items and accidents in the prevailing educational policy. They are, it is true, taught to the children, and the translation of them into their equivalents in the more conventional standards is explained and even practised. But they are certainly not made the usual and predominating system of calculation. The schools, to use a current phrase, do not provide a metric "atmosphere." Indeed in this respect their influence is entirely opposed to the spread of the metric system. No habit of thought or language will ever be generated by a practice or method which demands an act of translation. Facility in the performance of such an act may no doubt be cultivated by the influence of examinations and other artificial conditions; but these once removed the natural mental tendency promptly asserts itself and the spoken and written tongue is the one in which the individual is in the habit of thinking. It is, therefore, essential if the metric system is ever to come into general use that it be enforced as the common habit of school life so that children may be accustomed from the outset to think in its terms. These must be presented and adopted as the natural accents of the mother-tongue and not as the symbols of a foreign language. Such a policy would necessarily mean in the course of a few years the gradual substitution of the decimal system for the standards now in general use.

The same principles apply to the use of metric terms in the practice of medicine and pharmacy. No practical progress is being made in this direction. The reason is not that there is opposition to the principle of the proposed change. On the contrary, both educational experts and commercial authorities alike applaud the suggestion. But no essential advance is made because during the period of education though the metric system is presented as the ideal method, it is continually in practice pushed into a secondary position, and is not adopted even by those who testify to its advantages. It is idle to expect the practitioner to write his prescriptions in metric terms unless as a student he has been trained to think in these terms. And notoriously at present this is not the case. The metric system, though treated to empty scholastic compliments, is steadily avoided in all applications of educational discipline. Its praises are on the lips of every teacher. But this is not true of its practice. In these circumstances there is no wonder that the movement for reform halts. If teachers and authors and examiners would live up to their professions in this respect a new state of matters would promptly arise. Unfortunately, they receive no effective support from a quarter from which support might reasonably have been expected. The authorities responsible for the compilation of the British Pharmacopœia do, it is true, extend their recognition to the metric system and adopt it as an alternative to the present standards in various pharmaceutical operations. But they fail to lend it any countenance in the direction in which of all others their support would be valuable. That is to say the official doses are all stated in the weights and measures of the Imperial notation. As an inevitable consequence of this policy the various text-books of materia medica and therapeutics adopt the same notation, and thus the student during his period of pupilage becomes accustomed to think of doses in the current phraseology. Naturally in practice he continues the habits of his earlier years. Had the authorities adopted the policy of expressing the official doses in metric terms, retaining also for a time the present fashion so as not unduly to force the pace, all this might have been altered. Text-books and teachers would have had to accommodate themselves to the official tongue. Consequently the student would have learned to think and to speak in that tongue. And from this would follow as a natural result the writing of prescriptions in metric terms. It is along this line that reformers must hope for the success of their ambitions. Even the Jupiter of the House of Lords cannot drive people habitually to write and speak a language which is not the customary instrument of their thought.

